# l-Amphetamine improves poor sustained attention while d-amphetamine reduces overactivity and impulsiveness as well as improves sustained attention in an animal model of Attention-Deficit/Hyperactivity Disorder (ADHD)

**DOI:** 10.1186/1744-9081-4-3

**Published:** 2008-01-23

**Authors:** Terje Sagvolden, Tong Xu

**Affiliations:** 1 Institute of Basic Medical Sciences, Department of Physiology, University of Oslo, PO Box 1103 Blindern, NO-0317 Oslo, Norway; 2 Department of Pediatrics, Changzheng Hospital, The Second Military Medical University, Shanghai, China

## Abstract

**Background:**

ADHD is currently defined as a cognitive/behavioral developmental disorder where all clinical criteria are behavioral. Overactivity, impulsiveness, and inattentiveness are presently regarded as the main clinical symptoms. There is no biological marker, but there is considerable evidence to suggest that ADHD behavior is associated with poor dopaminergic and noradrenergic modulation of neuronal circuits that involve the frontal lobes. The best validated animal model of ADHD, the Spontaneously Hypertensive Rat (SHR), shows pronounced overactivity, impulsiveness, and deficient sustained attention. While dopamine release is decreased in SHR, norepinephrine concentrations are elevated. The primary objective of the present research was to test effects of a range of doses of the catecholamine agonists d- and l-amphetamine on ADHD-like symptoms in SHR.

**Methods:**

The present study tested behavioral effects of 0.64 to 1.91 mg/kg d-amphetamine; and 1.27 to 3.81 mg/kg l-amphetamine base/kg i.p. in male SHRs and their controls, the Wistar Kyoto rat (WKY). ADHD-like behavior was tested with a visual discrimination task measuring overactivity, impulsiveness and inattentiveness.

**Results:**

The striking impulsiveness, overactivity, and poorer sustained attention during baseline conditions in the SHR were improved by treatment with the amphetamine isomers. The dose-response curves were, however, different for the different behaviors. Most significantly, d-amphetamine reduced overactivity and impulsiveness more efficiently than comparable doses of l-amphetamine. The lowest dose of d-amphetamine and low-to-medium doses of l-amphetamine improved sustained attention. The highest dose of d-amphetamine used interfered with SHR behavior. A second study showed that the impaired sustained attention (percent correct lever choice) in the SHR was not due to impaired visual functions or poorer working memory.

**Discussion:**

The present results indicate that overactivity and impulsiveness may to some extent be associated with imbalances in neural circuits that differ from those causing poor sustained attention and that the two amphetamine isomers may affect the different neuromodulators differently. While d-amphetamine improved SHR overactivity, impulsiveness as well as sustained attention, the behavioral effects of l-amphetamine were relatively more specific for improving sustained attention than for the other 2 symptoms. Thus, while d- and l-amphetamine affect similar neuronal systems their relative potencies may be different.

## Background

Attention-deficit/hyperactivity disorder (ADHD) is currently defined as a cognitive developmental disorder where all clinical criteria are behavioral [1]. Overactivity, impulsiveness, and inattentiveness are presently regarded as the main clinical symptoms.

There have been many attempts to explain the origins of ADHD symptoms. A dual-process theory [2-5] suggests that less efficient reinforcement processes and deficient extinction of previously reinforced behavior may explain behavioral changes often described as response disinhibition [6] or poor executive functions [7].

ADHD is highly heritable and the genetic and neurobiological causes are likely to reside in brain catecholamines (for a review see [4]). Most likely, ADHD symptoms are associated with reduced post-synaptic efficacy of dopaminergic and noradrenergic modulation of neuronal circuits that involve the frontal lobes [8,9]. Imaging of striatal neuronal networks indicates reduced dopamine efficacy in ADHD [10]. Further, noradrenergic systems are involved in attention processes and prime prefrontal areas for response to sensory stimuli [11]. It is therefore not surprising that amphetamines and other catecholamine agonists have been the drugs of choice in medication of ADHD [8,9,12-14].

Recent neuropharmacological studies have shown that d- and l-amphetamine may affect electrically stimulated dopamine and norepinephrine release differently [15]. Thus, the two amphetamine isomers may affect the various receptors and neuromodulators of the central nervous system differently.

The spontaneously hypertensive rat (SHR) is the best validated animal model of ADHD. These rats show hyperactivity, impulsiveness and deficits in sustained attention [9,16-18]. The control strain is usually the Wistar Kyoto Rat (WKY) as this rat is the progenitor strain and its behavior is closely similar to that of other strains when tested in well-controlled operant tasks [17]. Drugs used in the pharmacological treatment of ADHD, usually catecholamine agonists have been shown to reduce ADHD-like behavior in this model [16,19-21]. The primary objective of the present research was to test effects of a range of doses of the catecholamine agonists d- and l-amphetamine on ADHD-like symptoms in SHR. It is predicted that d-amphetamine in particular should improve the ADHD-like behavior of the SHR.

## Study 1: Comparison of amphetamine isomers on sustained attention, overactivity and impulsiveness

The present study investigated behavioral effects of d- and l-amphetamine in an animal model of ADHD.

## Methods

### Subjects

A total number of 32 male rats, 16 SHR and 16 WKY, participated in this study. At the start of testing following 8 days acclimatization, the rats were 5 wk old and experimentally naïve. Young rats were required, as ADHD primarily is a child and adolescent disorder. The SHRs were obtained from Charles River Italy (SHR/Crl Ico) and the WKYs from Charles River France (WKY/Nico).

At the University of Oslo, the rats were housed individually in 41 × 25 × 25 (height) cm transparent cages and had free access to food (RM3 (E) from Special Diet Services, Witham, Essex CM8 3AD, UK). The rats had access to water at all times before the habituation session. Starting following completion of the habituation session, the rats were deprived of water for 21 hr a day; this is a moderate, but sufficient deprivation for motivating the animal. The temperature in the housing area was ~22°C. The light was on from 0700 to 1900 hours. The behavioral training took place between 1000 and 1330 hours seven days a week. The study was approved by the Norwegian Animal Research Authority (NARA), and was conducted in accordance with the laws and regulations controlling experiments/procedures in live animals in Norway.

### Behavioral apparatus

Sixteen Campden Instruments operant chambers were used in the study. The animal working space in eight of the chambers was 25 × 25 × 30 (height) cm and 25 × 25 × 20 (height) cm in the other eight chambers. A fan producing a low masking noise and the 2.8-W house light were on during the entire experimental session.

During training sessions, either one or both retractable levers were used (below). A 2.8-W cue light was located above each lever. The rats' response consisted of pressing one of the levers with a dead weight of at least 3 g to activate a micro-switch. The reinforcers (0.01 ml tap water) were delivered by a liquid dipper located in a small recessed cubicle with a 2.8-W cue light that lit up when a reinforcer was presented. A 7 × 5 cm transparent plastic lid separated the cubicle from the rat's working space. The rat could easily open the lid with a light push with the nose or paw. Each chamber was ventilated and placed in a sound-resistant outer housing. A computer and an online system (SPIDER, Paul Fray, Ltd., UK) recorded the behavior and scheduled reinforcers (drops of water).

Before the initiation of the study, the rats were assigned a chamber (1 through 16) and time of testing (1000 or 1200 hours) in a randomized and balanced way. The rat was returned to its living cage after each session and immediately given free access to water for 90 min.

### Response acquisition

The training period started with a single 30-min habituation session. During the habituation session, the lid between the working space and the reinforcement cubicle was kept open. The house light was on, but no lever was present, no cue light above any lever was lit and water was not delivered.

The habituation session was followed by two 30-min dipper training sessions. The lid was taped open, no levers were present, and the house light was on, but the cue lights above the levers were not lit. The computer delivered water on the average every 10 s independent of the rat's behavior (a variable-time schedule). Each water delivery was accompanied by the turning on of the cue light in the small recessed cubicle.

In the next two sessions, the rat was trained to open the lid to gain access to the water. The lid was not taped open, no levers were present and the lights above the levers were not activated. The house light was on. Each lid opening was followed by a presentation of a single drop of water. The cue light in the recessed cubicle was turned on when water was present.

During the subsequent two sessions, lever responding was shaped by the method of successive approximations [22]. During the first of these sessions, the rats learned to press the left lever in order to receive a reinforcer immediately following every press. The cue light above the left lever was now lit the entire session. The right lever was retracted into the wall and the light above the right lever was off. On the second session, the right lever was activated and the left lever retracted. During this session the light above the right lever was lit the entire session. The house light was on during both sessions. Following this shaping procedure, the animal had acquired the appropriate lever-pressing behavior.

From now on, both levers were present. The light above the levers shifted randomly. The light stayed lit above a lever for as long as it was the correct lever. This was the discriminative stimulus showing the rat which lever it had to press in order to receive a reinforcer. A concurrent extinction schedule was present on the wrong lever. There was never any light above the extinction lever. Thus, the present task was a simultaneous visual discrimination task. The first four of these sessions lasted for 30 min and the reinforcers were delivered following every correct lever press. Then followed a single session when the reinforcers were delivered according to a 15-s random-interval schedule. Whenever an interval had elapsed, the reinforcer was delivered immediately following the first correct response.

### Final schedule

The simultaneous visual discrimination task was used for testing effects of the drugs. An unpredictable 180-s random-interval schedule was in effect for 90 min on the correct lever (signaled by a constantly lit cue light above this lever) from session 13 on until the study was finished. Inter-reinforcer times ranged from 6 to 719 s in a randomized fashion with a skewed distribution modeled after the "Harvard golden tape" [23]. There was neither any external stimulus signaling that a reinforcer was programmed, nor any external stimulus signaling the time since the last response. A concurrent extinction schedule (never associated with any cue light) was present on the wrong lever. The house light was lit the entire session.

### Behavioral measures

Each session was divided into five 18-min segments (parts) in order to monitor intra-session changes in the behavior. For each segment, total number of presses on the correct and incorrect lever as well as number of reinforcers delivered were recorded. Time between consecutive correct responses (inter-response time, IRT) was also recorded.

The total number of lever presses is an expression of the general activity level and therefore a measure of degree of *activity*. The percent choice of the correct lever when the reinforcers are delivered infrequently is a measure of *sustained attention*. The number of responses with short IRTs (<0.67 s) is used as a measure of degree of *impulsiveness *(cannot hold back a response even when one knows it is an unnecessary one).

### Drug administration

Administration of the drugs started at session 49 when the behavior had stabilized. The two isomers of amphetamine were compared with vehicle and with each other. All rats received d-amphetamine sulphate and l-amphetamine sulphate. The dosing was balanced in the initial schedule. The schedule was later extended to take into account results obtained by session 76 (Drug day 9). This involved repeating some of the previous results when the previous ones had deviated from what was predicted and adding new doses that the obtained data suggested might be of interest: 0.64 mg/kg d-amphetamine and 1.27 mg/kg l-amphetamine. Each rat was injected intraperitoneally at a dose volume of 1 ml/kg body weight of the animal ~30 min before testing, with either vehicle (physiological saline) or drug. Drugs were administered every 3^rd ^or 4^th ^day. All rats received all doses according to a balanced design.

### Drugs

D-amphetamine sulphate (Lot 031298) and l-amphetamine sulphate (Lot FB-101-57) were supplied from Boeringer-Ingelheim US. Doses were 0.64, 1.27, and 1.91 mg/kg for d-amphetamine; and 1.27, 2.54, and 3.81 mg/kg for l-amphetamine. Doses were calculated as the weight of base using a conversion factor of 1.360 mg sulphate salt as equivalent to 1.000 mg base. Doses were based on pilot studies. Dosing solutions were prepared as a solution in physiological saline. Stock solutions, 1.91 mg/kg for d-amphetamine; and 3.81 mg/kg for l-amphetamine, were prepared at the start of the dosing period and kept at +4 to +6°C when not in use. Dilutions of the stock solutions were made each day of dosing.

### Data management and statistical procedures

The mean behavior was regarded as the drug response, and dose-response curves were plotted for each drug and strain. The data were processed by univariate and multivariate analyses of variance (ANOVAs and MANOVAs, respectively) with the Statistica 7.1 program [24]. Isomer and dose are within-subject variables. Strain is a between-subject variable. One control rat was identified as a statistical outlier with Grubbs' Test [25,26]. Post-hoc comparisons following MANOVAs were performed by the Unequal N HSD procedure, a generalization of Tukey's test to the case of unequal samples sizes (see [27], p. 975).

## Results

### General

Compared to WKY controls, SHRs showed pronounced overactivity, impulsiveness and poorer sustained attention. The drugs gave clear dose-response curves in the SHRs. The dose-response curves were different for the different behaviors. Low to medium doses of d- and l-amphetamine improved sustained attention. SHR overactivity and impulsiveness were reduced more by d-amphetamine than by l-amphetamine. Following the highest dose, d-amphetamine interfered with SHR behavior during the first part of the sessions.

### Acquisition

As is the case in children with ADHD [28,29], the symptoms developed with time, but differently for the different behaviors [18]. The final schedule was installed on session 13. A pronounced overactivity was seen in SHRs from this session on (see Additional files 1 and 2). SHR impulsiveness, responding within 0.67 s since the previous lever press although such a lever press was almost never reinforced, continued to increase in the SHR throughout the entire study [18]. This measure was accompanied by increased variability over days during the course of the study, something that is typical in ADHD [30-32]. Impulsiveness was subjected to a log10-transformation in order to obtain the more equal variances required by the ANOVAs.

#### Effects of drugs

##### Overactivity

**Figure 1:**
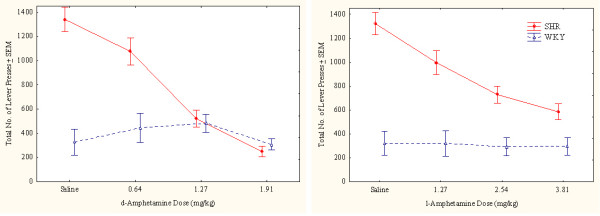
Effects of d-amphetamine (left) and l-amphetamine (right) on total number of lever presses (correct plus incorrect) by SHR and WKY controls. Means ± SEM.

**Figure 2:**
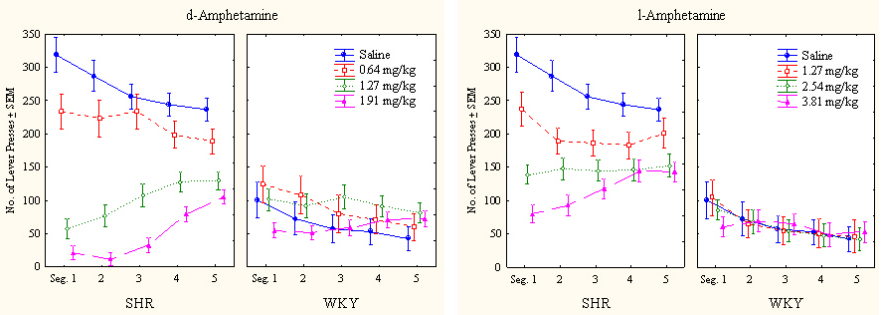
Within-session effects of d-amphetamine (the two panels to the left) and l-amphetamine (the two panels to the right) on number of lever presses (correct plus incorrect) by SHR and WKY controls. Means ± SEM.

The pronounced SHR overactivity was reduced by both drugs (Figure 1). Following the highest doses of either drug, the general activity level of the SHR approached that of the WKY, more so after the highest doses of d-amphetamine than after l-amphetamine. The highest dose of d-amphetamine reduced SHR behavior early in the session below WKY levels. This result indicates severely drugged behavior in the SHR (Figure 2). WKY behavior was unaffected by the drugs at these doses.

The ANOVA showed a statistically significant main effect of strain (*F(1,28) = 28.52, p < 0.001*). The MANOVA showed statistically significant interactions between strain × drug (*F(1,28) = 28.44, p < 0.001*), strain × dose, and strain × drug × dose (*Fs(3,26) > 5.78, ps < 0.004*). The 3-way interaction indicating that d-amphetamine reduced SHR hyperactivity more efficiently than l-amphetamine following the doses used. Post-hoc comparisons showed that all three doses of both d- and l-amphetamine reduced SHR overactivity (*ps < 0.002*). No dose or drug altered WKY activity level (*ps > 0.3*).

##### Impulsiveness

**Figure 3:**
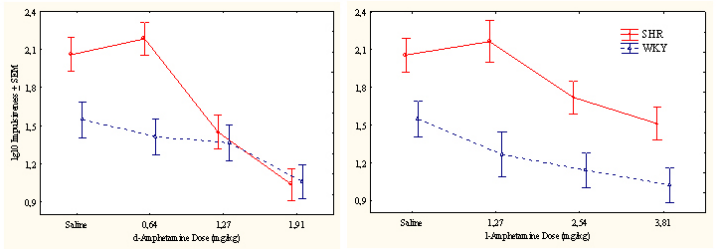
Effects of d-amphetamine (left) and l-amphetamine (right) on impulsiveness, responding within 0.67 s following the previous lever press, of SHR and WKY controls following log10 transformation. Means ± SEM.

**Figure 4:**
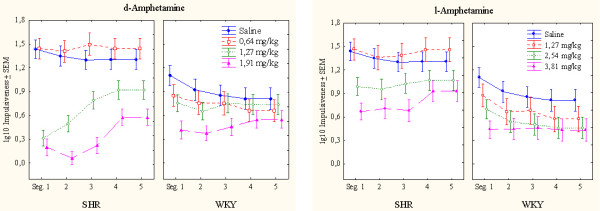
Within-session effects of d-amphetamine (the two panels to the left) and l-amphetamine (the two panels to the right) on impulsiveness, responding within 0.67 s following the previous lever press, of SHR and WKY controls following log10 transformation. Means ± SEM.

SHRs showed a pronounced impulsiveness that was reduced by both drugs. Following d-amphetamine, SHR impulsiveness was more affected than WKY impulsiveness. l-Amphetamine, however, produced similar effects in both strains (Figure 3). Except for the two highest doses of d-amphetamine producing more pronounced effects early in the session, particularly in SHRs, the other doses produced fairly stable effects throughout the session (Figure 4).

The ANOVA showed a statistically significant main effect of strain (*F(1,28) = 7.79, p < 0.01*). The MANOVA showed statistically significant interactions between strain × drug (*F(1,28) = 18.07, p < 0.001*), strain × dose, and strain × drug × dose (*Fs(3,26) > 6.54, ps < 0.002*). The 3-way interaction indicating that d-amphetamine reduced SHR impulsiveness more efficiently than l-amphetamine following the doses used. Post-hoc comparisons showed that the two highest doses of both d- and l-amphetamine reduced SHR impulsiveness (*ps < 0.005*). The highest d-amphetamine dose and the two highest l-amphetamine doses reduced WKY responding (*ps < 0.001*).

##### Sustained attention

**Figure 5:**
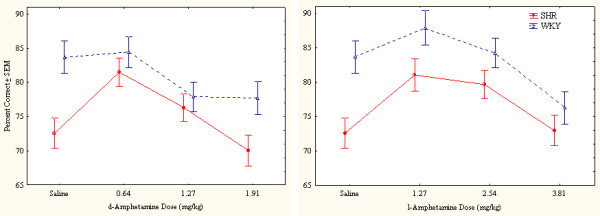
Effects of d-amphetamine (left) and l-amphetamine (right) on sustained attention, choice of the correct lever in percent of all lever presses, by SHR and WKY controls. Means ± SEM.

Without medication, SHRs showed poorer sustained attention than WKY controls. In contrast to the effects on activity and impulsiveness, where the most pronounced effects were seen following the highest doses of d-amphetamine, sustained attention appeared to improve in the SHR following low-to-medium doses only (Figure 5).

**Figure 6:**
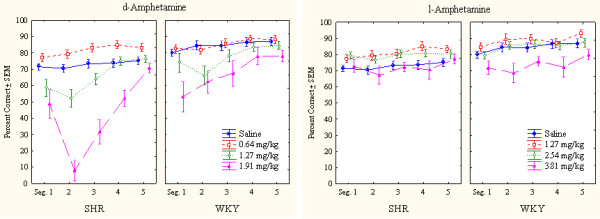
Within-session effects of d-amphetamine (the two panels to the left) and l-amphetamine (the two panels to the right) on sustained attention, choice of the correct lever in percent of all lever presses, by SHR and WKY controls. Means ± SEM.

The ANOVA showed a statistically significant main effect of strain (*F(1,28) = 5.25, p < 0.03*). The MANOVA showed no strain × drug, nor any 3-way strain × drug × dose interaction (*Fs(1,28) < 2.3, ps > 0.1*). There was however, a strain × dose interaction (*F(3,26) > 6.17, p < 0.003*). These results indicate that both drugs affected sustained attention similarly with the doses used. A more detailed within-session analysis showed however that especially the highest dose of d-amphetamine disrupted behavior early in the session (Figure 6) while there was no such disruption following l-amphetamine. Post-hoc comparisons showed that the 0.64 mg/dose of d-amphetamine and the 1.27 and 2.54 mg/kg doses of l-amphetamine significantly improved sustained attention in SHR (ps < 0.001). These improvements lasted the entire session (Figure 6). No dose or drug improved sustained attention in WKY, but the 3.81 mg/kg l-amphetamine made it significantly worse (p < 0.001).

##### Reinforcers delivered

The random-interval reinforcement schedule used was programmed so that even large individual differences in lever pressing would result in approximately 6 reinforcers (drops of water) during each 18-min segment of the session, even for the case of the less active strain. A major advantage of such a schedule is the fact that systematic strain differences in thirst should not be of concern when interpreting the other data.

**Figure 7:**
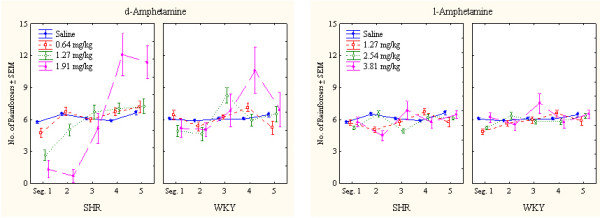
Within-session effects of d-amphetamine (the two panels to the left) and l-amphetamine (the two panels to the right) on number of reinforcers delivered to SHR and WKY controls. Means ± SEM.

The results show that both strains in general received 6 reinforcers each segment. However, following the 1.91 mg/kg d-amphetamine dose, the SHR strain was unable to maintain a sufficient response output during the initial 36 min to deliver the 12 reinforcers programmed (Figure 7). These reinforcers were, however, delivered during the last 36 min, thereby in effect producing an unintended RI 90 s schedule of reinforcement.

##### Stereotypy and severely drugged behavior

The 1.91 mg/kg d-amphetamine dose apparently produced a severely drugged behavior in the SHR during the two initial 18-min segments of the session. l-Amphetamine, on the other hand, never produced such an effect in any of the animals.

## Discussion

Study 1 investigated behavioral effects of d- and l-amphetamine on activity, impulsiveness and sustained attention in an animal model of ADHD. Overall, SHR behaviors were improved. The amphetamines affected these behaviors differently. The results showed clearer dose-response curves in the SHR than in the WKY strain. d-Amphetamine was more than twice as potent as l-amphetamine in reducing SHR hyperactivity and impulsivity. Low-to-medium doses of d- and l-amphetamine, improved sustained attention in the SHR while the highest dose did not. The highest doses reduced hyperactivity and impulsiveness. It is, however, likely that the highest dose of d-amphetamine caused severely drugged behavior in the SHR during the initial half of the session. Thus, only low-to-medium doses produced what might be regarded as real improvements in behavior.

## Study 2: No visual discrimination or memory problems in the SHR

Study 1 showed reduced percentage choice of the correct lever in SHRs when the reinforcers were delivered according to a random-interval 180 s schedule lasting 90 min (see also Figure 2 in [18]). Results of previous studies [33,34], indicate that there is an impaired sustained attention in the SHR. The main objective of Study 2 was to substantiate that the reduced percent correct lever choice in the SHR is due to poorer sustained attention rather than to poorer visual functions or problems with remembering the presentation of the discriminative stimulus (i.e., poorer "working memory").

### Procedure

#### Subjects

Eight experimentally naïve spontaneously hypertensive (SHR/NHsd) and 8 Wistar/Kyoto (WKY/NHsd) served as subjects. They were ~40 days old, weighted 170–250 g, at the start of the study. The animals were obtained from the Harlan UK Ltd., Bicester, England. Feeding and housing were similar to that in Study 1.

#### Behavioral apparatus

Eight of the Campden Instruments operant chambers from the previous study were used.

Before initiation of the study, the rats were assigned a chamber (1 through 8) and time of testing (0830 or 1000 hours) in a randomized and balanced way. They were run Mondays through Fridays. The rat was returned to its living cage after each session and immediately given free access to water for 45 min.

#### Response acquisition

The initial training was similar to that used in Study 1. A single 30-min habituation session was followed by two 30-min dipper training sessions and two sessions when water was delivered whenever the rat opened the lid into the recessed cubicle. During the next session, responding on the left lever was shaped by the method of successive approximations [22]. This was followed by two 30-min sessions stabilizing responding by reinforcing each press on the left lever. The cue light above the left lever was now lit the entire session. The right lever was not available during these sessions. During the next session, responding on the right lever was shaped in the same way as responding on the left. Then, the light above the right lever was lit the entire session. Following this shaping procedure the animal had acquired the appropriate behavior.

From now on, both levers were present. The discriminative stimulus light above the levers shifted randomly following each trial showing the rat which lever it had to press to obtain a reinforcer. The rat received the reinforcer immediately after pressing the correct lever. A concurrent extinction schedule, never associated with the cue light or a reinforcer, was present on the wrong lever.

After response acquisition, a 15-s random-interval schedule was in effect on the signaled correct lever for fifteen 21-min sessions in order to stabilize behaviors before start of the experiment.

#### Final schedule

Visual functions and working memory were tested during the next 62 sessions each lasting 24 min. A fixed ratio 1 schedule was in effect on the correct lever during these sessions. During this schedule, a reinforcer is delivered following every correct response.

There was no light above any of the two retracted levers at the start of a trial. Then the discriminative stimulus light above one of the two levers was lit for 5 s showing that this lever was going to be the correct one during this trial. Both levers were presented at the same time. Initially, levers were inserted 1 s following the cue light onset (the overlap between light and lever was 4 s) (Table 1).

**Table 1:** Experimental design of Study 2

Session no.	42–46	47–51	52–56	57–66	67–71	72–81	82–90	91–104
Overlap/delay	4 s	2 s	0 s	-2 s	-3 s	-4 s	-5 s	4 s

The light was turned off and both levers retracted immediately after the rat had pressed a lever. A reinforcer was delivered immediately if the rat had chosen the correct lever. Pressing the wrong lever never produced any reinforcer. After a 2 s intertrial interval, one cue light would be lit and the two levers would be presented again.

The time relation between presentation of the discriminative stimulus and lever presence was changed every five sessions until there was a 2 s delay from when the discriminative stimulus was turned off to insertion of the levers. Then extra sessions had to be given because the rats needed more training to reach a stable percentage correct lever choice (Table 1). The behavior during the final three sessions of stable performance of each discriminative stimulus-lever presence condition was used in the data analyses. Finally, 14 sessions were run with a 4 s overlap in order to check for any change in baseline behavior.

### Results

#### Percent correct

**Figure 8:**
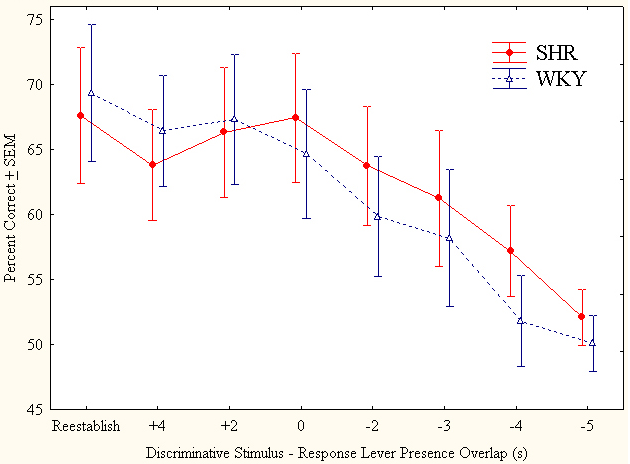
Choice of the correct lever in percent of all lever presses by SHR and WKY controls as function of decreasing overlap between the discriminative stimulus and the presentation of the levers. Means ± SEM.

The percent correct lever choice decreased in both SHR and WKY by decreasing overlap between the discriminative stimulus and the presentation of the levers (Figure 8). The MANOVA showed a significant main effect of this overlap *(F(7,8)= 16.10, p < 0.001)*, but neither any significant main effect of strain (*F (1,14) = 0.26, p > 0.5*), nor any interaction involving strain *(ps > 0.1)*.

#### Total number of trials

The total number of trials during the 24-min session decreased in both SHR and WKY by decreasing overlap between the discriminative stimulus and response lever presence. There was neither any significant main effect of strain (*F(1,14) > 0.8, p > 0.3*), nor any interaction involving strain *(ps > 0.1)*.

#### Reestablishment of baseline

Baseline behavior was successfully reestablished. The MANOVA showed no significant within-strain effects when baseline (the first five sessions) and reestablishment (the last ten sessions) were compared: the percent correct lever choice *(ps > 0.5)*, and the number of correct responses, *(ps > 0.3)*.

### Discussion

There was no strain difference in percent correct lever choice when the levers were made available during the presence of the discriminative stimulus. This result means that SHR had no visual problems. Further, both strains started at the same baseline and the percent correct lever choice declined at the same rate by decreasing overlap between the discriminative stimulus and the presentation of the levers (Figure 8). Thus, information the rats used for choosing the levers decayed at the same rate in SHR as in WKY control rats. Finally, the successful reestablishment of baseline behavior at the end of the study showed that the relation between percent correct lever choice and time from turning off the discriminative stimulus to the presentation of the levers was not related to the number of sessions run, training, or aging.

In conclusion, Study 2 showed that the reduced percent correct lever choice in SHR when reinforcers are few as in the Study 1 as well as previously published studies [18,20] may be described as poorer sustained attention and not visual problems or problems with remembering the location of the discriminative stimulus (working memory).

### General discussion

ADHD is currently defined as a cognitive/behavioral developmental disorder where all clinical criteria are behavioral. Overactivity, impulsiveness, and inattentiveness are presently regarded as the main clinical symptoms [1]. These symptoms have been operationalized in a long series of translational research investigating ADHD behavior in children and animal models [29-32,35,36]. The present Study 2 showed that the reduced percent correct lever choice frequently observed in SHR [18,20], is due to poorer sustained attention, not to poorer visual functions or poorer working memory.

ADHD is highly heritable and the genetic and neurobiological causes are likely to reside in reduced postsynaptic effects of catecholamines on glutamatergic and GABAergic neurons [4]. These changes apparently cause less efficient reinforcement processes and deficient extinction of previously reinforced behavior [3-5].

Amphetamines and other dopamine agonists have been the drugs of choice in medication of ADHD [8,9,12-14]. The present research investigated behavioral effects of doses of d-and l-amphetamine isomers. The dose-response curves were different for the different behaviors. These drugs improved SHR behavior as predicted. Most significantly, low-to-medium doses of both amphetamine isomers improved sustained attention, medium-to-high doses of d-amphetamine reduced overactivity and impulsiveness more efficiently than medium-to-high doses of l-amphetamine.

Although there seems to be an altered dopamine receptor 1 and 5 (DRD1/DRD5) neurotransmission in the SHR within the frontal-striatal circuitry involved in motor control [37], the present results may imply that ADHD is associated with a more general monoamine dysfunction than just a dopamine dysfunction alone [4]. It has been suggested that aspects of ADHD behavior result from imbalances between noradrenergic and dopaminergic regulation of neural circuits that involve the prefrontal cortex [9,11,38,39]. The present results indicate that overactivity and impulsiveness, at least to some degree, are caused by imbalances in neural circuits that differ from those causing poor sustained attention and that the two amphetamine isomers may affect the different neuromodulators differently. In fact, while d-amphetamine showed effects that seem to be relatively specific to the SHR, in that it reduced overactivity, impulsiveness and improved sustained attention, l-amphetamine showed a relatively greater effect in improving sustained attention compared with its effect on reducing overactivity and impulsiveness. This is perhaps reminiscent of the effects of the alpha-2a adrenoceptor agonist guanfacine which produced quite similar effects in both SHR and WKY [20]. This conclusion may be in general agreement with recent neuropharmacological results showing that d- and l-amphetamine may affect electrically stimulated dopamine and norepinephrine release somewhat differently [15].

Finally, it is reason to believe that the inefficient reinforcement and extinction processes related to ADHD give rise to the variable and unpredictable behavior associated with ADHD [4]. Future studies of the dynamics of behavior [31,32] will tell us if the apparent normalization of arithmetic means seen in the present as well as other studies, is associated with normalization of the organization of behavior following medication.

In conclusion, overactivity and impulsiveness in SHR may primarily be associated with reduced efficacy of dopamine functions causing inefficient reinforcement and extinction processes [2-5], while the poorer sustained attention may to some extent be associated with reduced efficacy of norepinephrine (despite the increased norepinephrine release normally seen in the SHR [9,40]).

## Competing interests

This research was in part financially supported by Shire Pharmaceutical Development LTD, England (Company No. 2486738), Hampshire International Business Park, Chineham, Basingstoke, Hampshire RG24 8EP, Great Britain. The company had no role, however, in the presentation of the research. Data presentation, statistics, discussion and conclusions that are the authors' own responsibility.

## Authors' contributions

TS designed the studies, ran Study 1 and wrote the manuscript. TX ran pilot studies leading up to Study 1 and performed Study 2. Both authors approved the final manuscript.

## Supplementary Material

Additional file 1 The video shows a normal male WKY control rat performing the visual discrimination task. Click here for file

Additional file 2 The video shows a Spontaneously Hypertensive Rat (SHR) performing the visual discrimination task. The rat is overactive and inattentive. Click here for file
